# Decision-makings of individual innovative behaviors in different educational settings: a case study of university student innovation competitions in China

**DOI:** 10.3389/fpsyg.2025.1573799

**Published:** 2025-09-03

**Authors:** Jiao Feng, Guoshuai Sun

**Affiliations:** ^1^College of Information Management, Xinjiang University of Finance & Economics, Urumqi, China; ^2^College of Civil Engineering and Architecture, Xinjiang University, Urumqi, China

**Keywords:** educational setting, individual innovative behavior, social psychology, educational psychology, evolutionary game model, innovation competition

## Abstract

**Introduction:**

With the intensification of global competition and rapid technological advancement, fostering innovation capabilities among university students has become a focal point of attention across society. However, within higher education environments, various contextual factors may exert direct or indirect influences on students’ motivation and effectiveness in implementing individual innovative behaviors.

**Methods:**

To address this issue, the present study takes the rapid rise of university student innovation competitions in China as a background case and adopts a dual perspective from social psychology and educational psychology. A tripartite evolutionary game model is developed, involving individual students, universities, and competition organizers. The model is designed to examine how students make strategic decisions regarding innovation under varying contextual influences, and how these decisions are shaped by the actions of universities and organizers.

**Results:**

The findings reveal that supportive educational policies, innovation-oriented campus cultures, and well-designed competition incentive mechanisms significantly enhance students’ motivation and the effectiveness of their innovative efforts. Moreover, strategic support from universities and organizers plays a critical role in sustaining students’ long-term engagement in innovation activities.

**Discussion:**

By integrating psychological theories with evolutionary game modeling, this study provides novel insights into how contextual interactions shape individual innovation behavior. The results offer practical implications for optimizing educational environments and provide evidence-based guidance for policymakers and educators aiming to foster student-led innovation.

## 1 Introduction

**Figure 1:**
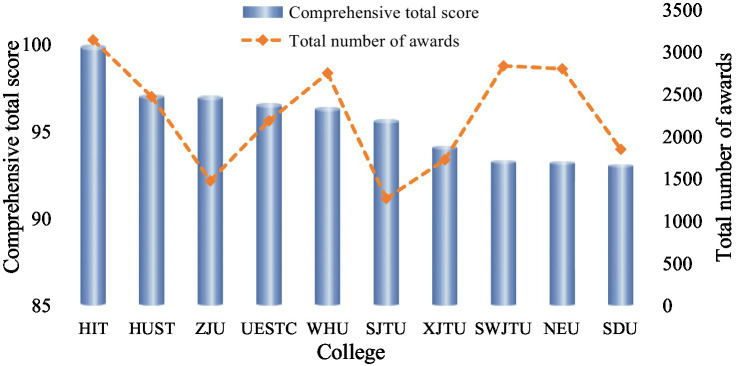
Statistics of university student competitions in China (2019–2023). The data source: *Annual Analysis Report on Student Competitions in Chinese Higher Education Institutions* published by the China Association of Higher Education.

In today’s era of globalization and rapid technological development, innovation capability has emerged as a core factor in national competitiveness. Particularly in the knowledge economy, the accelerated pace of technological advancements and industrial upgrades has driven a growing societal demand for innovative talents. As a crucial component of national innovation systems, university students’ development of innovation capabilities not only affects their individual career prospects but also contributes to national economic competitiveness and societal progress (Han et al., 2024; Mayhew et al., 2019). Fostering university students with innovation-driven mindsets and practical capabilities has become a focal point of higher education reform worldwide (May, 2023). In China, university student innovation competitions have flourished in recent years, becoming an essential innovation practice platform within the higher education system. In the context of China’s competitive higher education system, the intense pressure of university rankings has led many institutions to prioritize student innovation performance as an indicator of institutional excellence. This ranking-driven environment has created unique behavioral incentives and institutional strategies, making Chinese universities a representative and dynamic context for studying the mechanisms of students’ innovative behaviors. Statistics from educational authorities are shown in Figure 1. Therefore, using Chinese university student innovation competitions as research samples is representative and provides valuable insights for studying individual innovative behaviors in different educational settings. These competitions not only offer students opportunities to develop practical innovation skills but also provide universities and society with a pool of innovative talents (Zhao et al., 2020). They cover diverse fields, such as technological innovation and entrepreneurial competitions, attracting thousands of students to participate. In this context, the implementation of students’ innovative behaviors is not only influenced by individual traits and psychological processes but also closely related to external factors, such as the educational environment of universities and incentive mechanisms provided by competition organizers (Dai et al., 2022).

Although substantial research has explored the factors influencing innovative behaviors, most studies have focused on individual-level elements, such as cognition, motivation, and abilities (Amelink et al., 2019; Chou et al., 2023). However, the role of educational environments as external factors, particularly from the perspectives of educational psychology and social psychology, has yet to receive sufficient attention (Zhang et al., 2021). This gap limits our understanding of how to design more effective educational settings to promote innovation. Given that psychological challenges often intersect with other disciplines when addressing phenomena involving human thoughts and behaviors, integrating psychology with other fields has proven to be an effective approach (Roscoe et al., 2019; Feng and Chen, 2020). For instance, social psychology and educational psychology offer valuable frameworks for interpreting phenomena occurring in higher education settings (Steins et al., 2020; Seifried et al., 2024). In specific educational contexts, how to promote students’ implementation of innovative behaviors through various factors such as policy support, cultural atmosphere, and competition incentives remains an urgent question. This issue is particularly pertinent in China, where the growing popularity of university student innovation competitions may present complex interaction mechanisms between different educational environments and students’ innovative behaviors, warranting in-depth investigation.

Against this backdrop, this study poses a key question: how do educational environments influence university students’ decision-making regarding innovative behaviors in innovation competitions? Specifically, how can analyses of real-world cases from Chinese university innovation competitions, informed by educational psychology and social psychology, reveal the collaborative effects of different educational contextual factors on students’ innovation decisions and behaviors? This multifaceted and intricate question intersects various disciplines, including educational psychology, social psychology, and behavioral economics.

The primary objective of this research is to explore the mechanisms through which different educational contexts influence university students’ decision-making regarding innovative behaviors. Specifically, it seeks to examine how factors such as policy support, campus cultural atmosphere, and competition incentive mechanisms affect students’ motivation and effectiveness in implementing innovative behaviors. Through an in-depth analysis of real-world cases from Chinese university student innovation competitions, the study investigates the interactive relationships and decision-making mechanisms among students, universities, and competition organizers.

This study makes three major contributions. First, by integrating social psychology and educational psychology theories, it develops a novel tripartite evolutionary game model to reveal how educational environments influence students’ innovative behaviors through various strategic interactions. This model addresses the gap in existing literature regarding the impact of educational environments on university students’ innovative behaviors and extends the application of evolutionary game theory to the field of educational psychology. Second, through case analyses of Chinese university student innovation competitions, this study offers concrete policy recommendations for universities and competition organizers, guiding them to optimize educational environments and design innovation competitions that effectively stimulate students’ innovative potential. These findings not only hold significant reference value for China’s higher education system but also provide valuable insights for other countries’ innovation education initiatives. Third, by applying the tripartite evolutionary game model to analyze how interactions among various stakeholders in educational contexts influence university students’ innovation decision-making, the study reveals the dynamic evolutionary processes and key influencing factors in students’ decision-making under different educational environments. This model not only broadens the scope of evolutionary game theory applications but also provides new perspectives and methodologies for the study of innovative behaviors.

The structure of the remaining sections of this paper is as follows: Section 2 provides a literature review. Section 3 introduces the theoretical framework and hypotheses. Section 4 develops a three-party evolutionary game model. Section 5 discusses the behavioral decisions under different educational settings. Section 6 presents the discussion. Section 7 concludes with the findings and policy recommendations.

## 2 Literature review

### 2.1 Definition and relevant theories of innovative behavior

Innovative behavior has been conceptualized differently across disciplines. In psychology, it is often defined as the external expression of an individual’s creativity, including the generation and implementation of novel ideas (Amabile, 1988). In organizational management, innovative behavior extends to the introduction and application of new products, processes, and administrative practices that benefit the organization (Janssen, 2000). Within educational contexts, innovative behavior also encompasses the integration of cross-disciplinary knowledge and skills to solve practical problems in novel ways (Zhao et al., 2020). Thus, innovative behavior typically refers to an individual’s ability to respond to problems or challenges through creative thinking, resource integration, and the generation of novel solutions that drive development (Chen et al., 2018; Schumpeter, 1934). It is not limited to technological or product innovation but also encompasses improvements in work processes, teamwork, and management methods (Ozberk and Uzunboylu, 2017). Innovative behavior has significant implications for personal development, particularly in terms of career progression, job performance, and individual psychological well-being. Research on innovative behavior can be traced back to motivation theories in social psychology. Social cognitive theory suggests that external rewards can have dual effects on innovative behavior, either stimulating an individual’s creative motivation or suppressing innovation (Malek et al., 2020). Furthermore, Self-Determination Theory (SDT) emphasizes the core role of intrinsic motivation in innovative behavior, arguing that intrinsic motivation is the primary driver of an individual’s engagement in innovative actions (Fischer et al., 2019). Another closely related theory is the “multi-stage model,” which posits that innovative behavior is a process that typically unfolds in several stages, including problem identification, idea generation, selection, and execution (Wamba and Queiroz, 2022). This process is influenced by a variety of factors, including personal psychology, the external environment, and social support. Research has shown that individuals with high levels of innovative behavior often possess strong self-efficacy and intrinsic motivation (Lin, 2007). From a psychological perspective, attitudes toward innovation and innovation performance have a significant positive impact on innovative behavior (Zhang et al., 2021). By providing personal development support and guidance, individuals’ psychological resilience, innovative enthusiasm, and work efficiency can be enhanced (Liu, 2022). However, existing research has primarily focused on individual-level factors such as cognition, motivation, and self-efficacy, with limited structured analysis of how educational environments modulate these psychological mechanisms.

### 2.2 Impact of educational environment on innovation

The educational environment plays a critical role in stimulating students’ innovative behavior. The maintenance of an effective educational environment requires the joint efforts of all educational stakeholders, particularly teachers (Hammar Chiriac et al., 2024). Universities, as the cradle for cultivating innovative talents, have their internal policies, campus culture, and incentive mechanisms, which directly influence students’ innovation motivation and behaviors (Daradkeh, 2023). For example, university policies such as the inclusion of innovation-focused courses, support for research projects, and guarantees of academic freedom are important factors influencing students’ innovative behavior. Studies have shown that innovation-focused educational programs and research support provided by universities significantly enhance students’ creative thinking and practical abilities (Cao and Zhou, 2018). Policy support is not only reflected in resource allocation but also in institutional safeguards such as encouraging interdisciplinary collaboration and providing entrepreneurial guidance (Sandhu et al., 2015). The reward mechanisms for innovation activities within universities, such as scholarships and competition prizes, can also effectively increase students’ motivation for innovation (Tseng et al., 2020). In addition, campus culture, including the university’s values, cultural atmosphere, and the extent to which innovation is supported, is another crucial environmental factor that fosters innovative behavior. A campus culture centered on innovation can stimulate students’ creative potential (Yuan et al., 2022). The impact of organizational culture on innovation lies in its ability to regulate and incentivize members’ behaviors. Specifically, a campus culture that emphasizes free exploration, tolerance for failure, and encouragement of collaboration supports students in engaging in creative activities (Zhu and Engels, 2014). Conversely, an excessively competitive and utilitarian-oriented campus culture may suppress students’ willingness to innovate (Draper et al., 2021). Furthermore, incentive mechanisms play an important role in motivating students’ innovative behavior. Self-Determination Theory (Deci and Ryan, 1985) emphasizes that external rewards that align with students’ intrinsic motivation can enhance their innovative behavior. However, excessive external control may undermine students’ creative thinking (Deci and Ryan, 2008; Lanford et al., 2019). In innovation competitions and similar activities, effective incentive mechanisms should include not only material rewards but also recognition and growth opportunities at a psychological level (Yan et al., 2018). For instance, teamwork and mutual support among peers can also play an important role in the innovation process (Zhao et al., 2020). Nevertheless, the current literature remains largely descriptive, emphasizing isolated factors rather than offering clear models of interaction among educational subsystems. Moreover, how educational environments influence student behavior in varied temporal and spatial contexts has yet to be systematically explored.

### 2.3 Application of evolutionary game theory in social and educational psychology

Evolutionary game theory (EGT), first introduced by one of the founders of game theory, Nash (1950), is based on analyzing the dynamic process of interactions and strategic choices among individuals to study behavioral evolution (Gintis, 2000). In social psychology and educational psychology, evolutionary game models have been widely used to understand individual decision-making, particularly in contexts such as collective action, cooperation and competition, and innovative behavior (Itao and Kaneko, 2025). Regarding the application of evolutionary game models to innovative behavior, the model was used to study the strategy choices of team members in the process of collaborative innovation (Zhao and Zou, 2025). Their findings suggest that cooperative behavior tends to be stable within a group. However, in highly competitive environments, an individual’s innovative behavior is often influenced by external incentive mechanisms. Evolutionary game theory can effectively reveal the decision-making and strategy evolution of students in innovation competitions under different educational environments. In educational contexts, evolutionary game models are commonly used to analyze the interactions among educational policies, teacher behavior, and student behavior. Through game-theoretic analysis, the cooperation and competition patterns among students, teachers, and educational institutions can be uncovered, and it can provide insights into how to design educational policies that promote students’ innovative behavior (Ma and Yang, 2024). For example, Chen et al. (2024) constructed an evolutionary game model involving both teachers and students to analyze how their interactions influence the effectiveness of innovation education. They found that educational policies and incentive mechanisms significantly affect the innovative behavior of both teachers and students, and inappropriate policies may reduce cooperation between teachers and students, thereby affecting the output of innovative behaviors. Although evolutionary game theory can dynamically simulate the evolution of individual behaviors and reveal the stability of different strategies in varying environments (Betz et al., 2024), one limitation is its assumption that participants’ decisions are rational and based on complete information. In actual educational settings, students’ innovative behaviors are often influenced by cognitive biases, emotions, and social interactions, which need to be considered further within the model (El Nemar et al., 2020). Although some scholars have applied game-theoretic models to teacher–student interactions, research remains scarce in the context of innovation education—particularly regarding student competition behavior—where student-centered models that integrate both institutional policies and external incentives are largely lacking.

Accordingly, this study integrates psychological drivers with strategic feedback mechanisms by adopting a tripartite game-theoretic perspective encompassing universities, students, and competition organizers. The proposed evolutionary game model not only fills a structural gap in existing modeling approaches but also aligns conceptually with educational psychology’s emphasis on the integration of social interaction and intrinsic motivation.

## 3 Theoretical framework and hypotheses

### 3.1 Three-party evolutionary game model

This study adopts a three-party evolutionary game model to analyze the interactions between individual students, universities, and competition organizers in different educational contexts, with the aim of revealing how these parties influence students’ decision-making regarding innovative behavior in varying educational environments. Evolutionary game theory, which originated in biological population behavior analysis, has been widely applied in social sciences, particularly in educational and innovation behavior research (Zhang, 2024). In this model, the three participants in the game are students (Player 1), universities (Player 2), and competition organizers (Player 3).

#### 3.1.1 Student (Player 1)

Students refer to individual agents making behavioral choices based on perceived costs, benefits, and motivational factors. In game-theoretic terms, they are boundedly rational actors whose strategies evolve through learning and adaptation.

As the individual participant in the game, students’ decisions directly determine whether they engage in innovative behavior. Students’ decisions are influenced by their intrinsic motivation, the educational environment (such as policy support, campus culture), and external incentives (such as competition reward mechanisms). In the game model, students have two main choices: one is to actively engage in innovative behavior, and the other is to refrain from innovation or adopt a conservative approach.

#### 3.1.2 University (Player 2)

Universities are institutional stakeholders that shape students’ choices through support policies, resources, and cultural context. They function as strategic agents aiming to optimize institutional outcomes.

Universities, as the providers of the educational environment, have the ability to influence students’ innovative behavior. The university’s decisions are reflected in its innovation education policies, resource allocation, and academic support. For instance, universities can encourage students’ innovative behavior by offering innovation-focused courses, providing research funding, and fostering interdisciplinary collaboration. In the game, the university’s choice is whether to provide supportive policies, such as innovation programs and academic guidance, or to neglect innovation support and adhere to more traditional educational models.

#### 3.1.3 Competition organizers (Player 3)

Competition organizers represent external entities (e.g., enterprises, government bodies) that design and implement incentive structures. In the game model, they act as initiators influencing the payoffs of other agents.

Competition organizers, as an external force within the educational environment, primarily influence students’ behaviors through the design of competition rules, reward mechanisms, and incentive policies. Competition organizers can motivate students to innovate by offering rewards, rankings, and scholarships. In interactions with universities and students, the strategies of competition organizers determine the level of support for students’ innovative behavior and their guidance during the competition.

In this three-party game, the interactions between the individual students, universities, and competition organizers are multi-level. Each party’s decisions not only affect their own rewards but also influence the decisions of the other participants through the game dynamics. The evolutionary game model emphasizes the process of dynamic evolution through the game, aiming to find stable strategy combinations that reveal how the design of the educational environment can foster continuous innovative behavior among students.

### 3.2 Hypotheses

Based on the literature review and theoretical framework, the following hypotheses are proposed for this study:

*H1*: There is a positive relationship between educational policy support and students’ innovative behavior.

According to Expectancy-Value Theory, students are more likely to engage in goal-directed behavior when they perceive the expected value of an activity as high and the associated cost as low (Wigfield and Eccles, 2000). Educational policies that support innovation, particularly in the form of innovative course offerings, resource allocation, and research funding, can effectively stimulate students’ innovative behavior. According to previous studies (Zhang and Chen, 2021), universities can enhance students’ motivation for innovation by implementing robust innovation-supporting policies, encouraging students to participate in innovation competitions and research projects. Therefore, the more universities invest in educational policies, the higher the likelihood of students engaging in innovative behavior.

*H2*: Campus cultural atmosphere positively influences students’ innovative behavior.

According to Social Cognitive Theory, environmental factors such as norms, role modeling, and cultural expectations play a critical role in shaping individual behavior (Bandura, 1986). Campus culture, as a key component of the educational environment, plays a crucial role in influencing students’ innovative behavior (Winks et al., 2020; Shahzad et al., 2024). A campus culture that supports innovation provides a free and inclusive environment that encourages students to express creative ideas and experiment. Conversely, a culture that is overly utilitarian or highly competitive may suppress students’ motivation to innovate. Thus, this study hypothesizes that there is a significant positive relationship between a campus’s innovation-supporting cultural atmosphere and students’ innovative behavior.

*H3*: Competition incentive mechanisms significantly impact students’ innovative behavior.

Based on Self-Determination Theory, external rewards can enhance intrinsic motivation when they are perceived as informational rather than controlling in nature (Manninen et al., 2022). Competitions, as a key driver of students’ innovative behavior, rely heavily on the design of their incentive mechanisms. Reward mechanisms, competition rules, and participation opportunities directly affect students’ motivation to engage in and perform innovatively (Lattuca et al., 2014). Both material incentives (such as prizes) and psychological incentives (such as recognition and opportunities for exposure) can significantly enhance students’ investment in innovation. Thus, a well-designed competition incentive mechanism can positively promote the implementation of students’ innovative behavior.

*H4*: There is an interaction effect between university support strategies and competition incentive mechanisms on students’ innovative behavior.

In organizational behavior research, Resource Synergy Theory suggests that coordinated support from multiple actors is more effective in promoting behavioral engagement than isolated incentive measures (Chen and Chen, 2013). Within the educational environment, the support strategies of universities and the incentive mechanisms of competition organizers are not independent; rather, there is an interaction effect (Park, 2012). While universities provide policy support and academic resources, competition organizers further promote students’ innovative behavior through reward and punishment mechanisms. The synergistic effect of both can maximize students’ motivation for innovation (Li et al., 2024). This study hypothesizes that the interaction between university policy support and competition incentives can more effectively foster students’ innovative behavior.

*H5*: Educational contexts have a time-effect on students’ decision-making regarding innovative behavior.

Motivational trajectory models in educational psychology suggest that prolonged exposure to a supportive environment gradually shapes behavioral tendencies over time (Wigfield et al., 2021). When deciding whether to engage in innovative behavior, students are influenced by the cumulative impact of the educational context over time. Policy support, cultural atmosphere, and incentive mechanisms in the educational environment do not have an immediate effect but accumulate over time, gradually influencing students’ decisions (Aoki et al., 2011; Hilmersson and Hilmersson, 2021). For example, a long-term innovation culture and stable policy support can lead students to gradually form a habitual approach to innovative behavior. Therefore, this study hypothesizes that the influence of the educational environment on students’ innovative behavior is significantly time-dependent and becomes more pronounced over time.

## 4 Model construction

This study adopts a three-party evolutionary game model to analyze the decision-making process of students’ innovative behavior, focusing on the interactions between individual students, universities, and competition organizers. The core assumption of the model is that each party adjusts its own behavior strategy based on the strategies chosen by the other two parties. The basic construction of the model is as follows.

### 4.1 Participants and strategy choices

In the model, the three participants make the following strategy choices:

- Student (Player 1): Choose either “Participation in Innovation” or “Non-participation in Innovation.”
- University (Player 2): Choose either “Support Innovation” or “Do Not Support Innovation.”
- Competition organizers (Player 3): Choose either “Incentivize Innovation” or “No Incentives.”

### 4.2 Payoff function and revenue calculation

Each participant’s payoff depends on their chosen strategy as well as the strategy combinations of the other participants. The following payoff functions are established. The payoff matrix for the three parties—competition organizers, individual students, and universities—is constructed as follows:

- Competition organizers: In practice, organizers can include government agencies, enterprises, industry associations, and non-profit organizations, each with distinct incentive structures and objectives. When students participate actively in the innovation competition, the performance of the competition organizers will be E₁ (if they choose to incentivize) or E₂ (if they choose not to incentivize). If the organizers choose the “Incentive” strategy, they incur an incentive cost of C₁. If students do not actively participate, the organizers face a negative outcome (F₁) due to performance evaluation from higher authorities. If students actively participate, the organizers will reward both the students and the universities with a total reward expenditure of W₁.
- Universities: If the university supports innovation, it incurs an incentive cost of C₂. If students actively participate and the university actively organizes, the university will receive rewards from the competition organizers, represented as *α*W₁, where α is the reward-sharing coefficient between the university and the student. Additionally, the university will receive extra rewards from the government for promoting innovation policies, represented as *γ*W₂, where γ is the reward-sharing coefficient between the university and the student. Furthermore, the university will need to spend W₃ to reward the student. However, if the university does not support innovation, but students still actively participate, the university will receive rewards from the competition organizers, represented as βW₁, and government reward revenue θW₂.
- Individual students: If individual students actively participate in the competition, they incur various costs, denoted as C₃. If the competition organizers incentivize and the university supports innovation, the student will receive the competition organizers’ reward, (1-*α*)W₁, the government’s extra reward, (1-*γ*)W₂, and the university’s reward, W₃. If the university does not support innovation, the student will receive the university’s reward of (1-*β*)W₁, and the competition organizers’ reward of (1-*θ*)W₂.

In summary, based on the above assumptions and payoff functions, the evolutionary game model for competition organizers, universities, and students is constructed. The corresponding payoff matrix is shown in Table 1.

**Table 1:** Payoff matrix of the competition organizer, university, and individual student.

Competition organizer	Individual student	University	
		Support	Non-support
Incentive	Participation	$E_{1}-C_{1}-W_{1}$	$E_{1}-C_{1}-W_{1}$
		$-C_{2}+\alpha W_{1}+\gamma W_{2}-W_{3}$	$\beta W_{1}+\theta W_{2}$
		$-C_{3}+(1-\alpha )W_{1}+(1-\gamma )W_{2}+W_{3}$	$-C_{3}+(1-\beta )W_{1}+(1-\theta )W_{2}$
	Non-participation	$-C_{1}-F_{1}$	$-C_{1}-F_{1}$
		$-C_{2}$	0
		0	0
Non-Incentives	Participation	$E_{2}$	$E_{2}$
		$-C_{2}+\gamma W_{2}-W_{3}$	$\theta W_{2}$
		$-C_{3}+(1-\gamma )W_{2}+W_{3}$	$-C_{3}+(1-\theta )W_{2}$
	Non-participation	$-F_{1}$	$-F_{1}$
		$-C_{2}$	0
		0	0

All parameters in the table are greater than 0.

### 4.3 Construction of replicator dynamic equations

#### 4.3.1 Competition organizer

Let the expected payoffs for the competition organizer when adopting the “Incentive” and “Non-Incentive” strategies be U_11_ and U_12_, respectively. The average expected payoff is $\bar{U_{1}}$ , which is given by Equations 1–3:

(1) $$U_{11}=\{\begin{array}{c} \mathrm{yz}(E_{1}-C_{1}-W_{1})+y(1-z)(E_{1}-C_{1}-W_{1})\\ +(1-y)z(-C_{1}-F_{1})+(1-y)(1-z)(-C_{1}-F_{1}) \end{array}$$

(2) $$U_{12}=\{\begin{array}{c} \mathrm{yz}(E_{2})+y(1-z)(E_{2})+(1-y)z(-F_{1})\\ +(1-y)(1-z)(-F_{1}) \end{array}$$

(3) $$\bar{U_{1}}=xU_{11}+(1-x)U_{12}$$

The replication dynamic equation for the competition organizer is given in Equation 4:

(4) $$\begin{array}{l} F(x)=\frac{\mathrm{dx}}{\mathrm{dt}}=x(1-x)(U_{11}-U_{12})\\ =x(1-x)(yE_{1}-\mathrm{yW}-1C_{1}-yE_{2}) \end{array}$$

**Figure 2:**
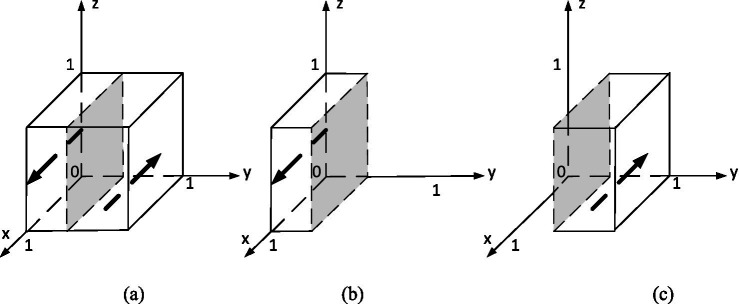
Evolutionary phase diagram of competition organizers’ strategies. $(a)\;y=y^{*}(b)\;y<y^{*}=(c)\;y>y^{*}.$

In order to analyze its evolutionarily stable strategy, based on the stability principle of differential equations, the strategy phase diagram for the competition organizer is shown in Figure 2. Where, $y^{*}=\frac{C_{1}}{E_{1}-W_{1}-E_{2}}$ _._

This equation describes how the proportion of competition organizers choosing to “incentivize” evolves over time. The change rate depends on the payoff difference between incentivizing and the average expected payoff. If the combined cost of incentivization is smaller than the performance gain, and if student and university participation are high, then more organizers will adopt the incentive strategy. Conversely, when participation is low or rewards are costly, the motivation to incentivize weakens.

#### 4.3.2 Student

Let the expected payoffs for the student choosing “participate” and “not participate” be U_21_ and U_22_, respectively, and the average expected payoff be $\bar{U_{2}}$ . These expected payoffs can be derived from Equations 5–7:

(5) $$U_{21}=\{\begin{array}{c} \begin{array}{l} \mathrm{xz}(-C_{3}+W_{1}-\alpha W_{1}+W_{2}-\gamma W_{2}+W_{3})\\ +x(1-z)(-C_{3}+W_{1}-\beta W_{1}+W_{2}-\theta W_{2}) \end{array}\\ \begin{array}{l} +(1-x)z(-C_{3}+W_{2}-\gamma W_{2}+W_{3})\\ +(1-x)(1-z)(-C_{3}+W_{2}-\theta W_{2}) \end{array} \end{array}$$

(6) $$U_{22}=\{\begin{array}{c} \mathrm{xz}(0)+x(1-z)(0)+(1-x)z(0)\\ +(1-x)(1-z)(0) \end{array}$$

(7) $$\bar{U_{2}}=yU_{21}+(1-y)U_{22}$$

The replication dynamic equation for the student is as follows:

(8) $$\begin{array}{l} F(y)=\frac{\mathrm{dy}}{\mathrm{dt}}=y(1-y)(U_{21}-U_{22})\\ \;\;\;=y(1-y)\{\begin{array}{c} -\mathrm{z\gamma}W_{2}+zW_{3}-C_{3}+W_{2}-\theta W_{2}+\mathrm{z\theta}W_{2}\\ +\mathrm{xz\alpha}W_{1}+xW_{1}-\mathrm{x\beta}W_{1}+\mathrm{xz\beta}W_{1} \end{array} \end{array}$$

**Figure 3:**
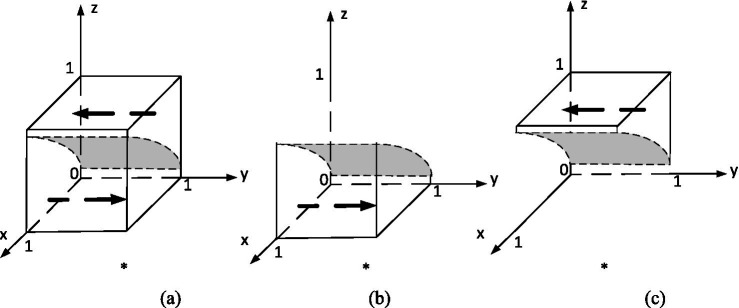
Evolutionary phase diagram of students’ strategies. $(a)\;z=z^{*}(b)\;z<z^{*}\;(c)\;z>z^{*}.$

In order to analyze its evolutionarily stable strategy, based on the stability principle of differential equations, the strategy phase diagram for the student is shown in Figure 3. Where, $z^{*}=\frac{C_{3}-W_{2}+\theta W_{2}-xW_{1}+\mathrm{x\beta}W_{1}}{-\gamma W_{2}+\theta W_{2}+\mathrm{x\alpha}W_{1}+\mathrm{x\beta}W_{1}}$ .

This equation models the evolution of students’ willingness to participate in innovation. The rate of increase in participation depends on the perceived net benefit: when students expect to receive substantial rewards from both organizers and universities and when the participation of other stakeholders is high, the incentive to engage grows. However, if the cost outweighs the expected benefits, participation declines. This mirrors behavioral economic principles, where students maximize utility under uncertainty and peer influence.

#### 4.3.3 University

Let the expected payoffs for the university choosing “support” and “not support” be U_31_ and U_32_, respectively, and the average expected payoff be $\bar{U_{3}}$ . These expected payoffs can be derived from Equations 9–11:

(9) $$U_{31}=\{\begin{array}{c} \mathrm{xy}(-C_{2}+\alpha W_{1}+\gamma W_{2}-W_{3})+x(1-y)(-C_{2})\\ +(1-x)y(-C_{2}+\gamma W_{2}-W_{3})+(1-x)(1-y)(-C_{2}) \end{array}$$

(10) $$U_{32}=\{\begin{array}{c} \mathrm{xy}(\beta W_{1}+\theta W_{2})+x(1-y)(0)+(1-x)y(\theta W_{2})\\ +(1-x)(1-y)(0) \end{array}$$

(11) $$\bar{U_{3}}=zU_{31}+(1-z)U_{32}$$

The replication dynamic equation for the university is given in Equation 12:

(12) $$\begin{array}{l} F(z)=\frac{\mathrm{dz}}{\mathrm{dt}}=z(1-z)(U_{31}-U_{32})\\ \begin{array}{cccc} & & & = \end{array}z(1-z)(\begin{array}{l} -\mathrm{xy\beta}W_{1}-\mathrm{y\theta}W_{2}\\ +\mathrm{xy\alpha}W_{1}+\mathrm{y\gamma}W_{2}-yW_{3}-C_{2} \end{array}) \end{array}$$

**Figure 4:**
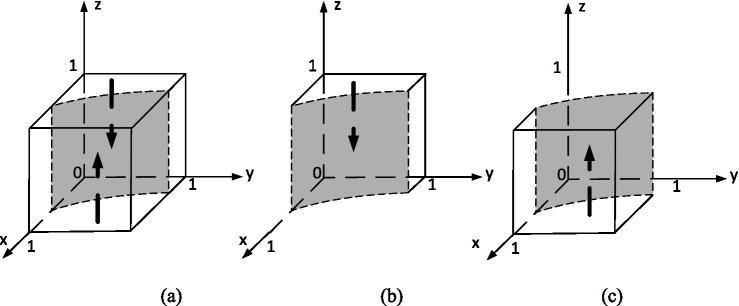
Evolutionary phase diagram of universities’ strategies. $(a)\;x=x^{*}(b)\;x<x^{*}\;(c)\;x>x^{*}.$

In order to analyze its evolutionarily stable strategy, based on the stability principle of differential equations, the strategy phase diagram for the university is shown in Figure 4. Where, $x^{*}=\frac{-\mathrm{y\theta}W_{2}+\mathrm{y\gamma}W_{2}-yW_{3}-C_{2}}{\mathrm{y\beta}W_{1}-\mathrm{y\alpha}W_{1}}$ .

This equation tracks how universities’ strategic choice to support innovation changes over time. Support is more likely when the expected rewards from organizers and government outweigh internal costs. High levels of student and organizer engagement further encourage support. Conversely, if universities perceive low returns or low external engagement, they are less likely to participate. This aligns with institutional theory, where policy decisions depend on resource exchange and external signaling.

Based on the psychological perspective, the game-theoretic analysis of university students’ participation in innovation competitions exhibits characteristics of asymmetric game players with information asymmetry. Therefore, the evolutionarily stable strategy is a pure strategy. In this context, we only need to discuss the asymptotic stability of the eight local equilibrium points: (0, 0, 0), (1, 0, 0), (0, 1, 0), (0, 0, 1), (1, 1, 0), (1, 0, 1), (0, 1, 1), and (1, 1, 1) when F(x), F(y), and F(z) are all equal to 0. From the aforementioned replicator dynamics equations for the three parties, the corresponding eigenvalues for each stable point can be obtained, as shown in Table 2.

**Table 2:** Eigenvalues of evolutionary game equilibrium points.

Equilibrium points	Eigenvalues		
	$\lambda_{1}$	$\lambda_{2}$	$\lambda_{3}$
(0, 0, 0)	$-C_{1}$	$-C_{2}$	$W_{2}-C_{3}-\theta W_{2}$
(1, 0, 0)	$C_{1}$	$-C_{2}$	$W_{1}-C_{3}+W_{2}-\beta W_{1}-\theta W_{2}$
(0, 1, 0)	$C_{3}-W_{2}+\theta W_{2}$	$\gamma W_{2}-W_{3}-C_{2}-\theta W_{2}$	$E_{1}-C_{1}-E_{2}-W_{1}$
(0, 0, 1)	$C_{2}$	$-C_{1}$	$W_{2}-C_{3}+W_{3}-\gamma W_{2}$
(1, 1, 0)	$C_{1}-E_{1}+E_{2}+W_{1}$	$C_{3}-W_{1}-W_{2}+\beta W_{1}+\theta W_{2}$	$\alpha W_{1}-W_{3}-C_{2}-\beta W_{1}+\gamma W_{2}-\theta W_{2}$
(1, 0, 1)	$C_{1}$	$C_{2}$	$W_{1}-C_{3}+W_{2}+W_{3}+\alpha W_{1}-\gamma W_{2}$
(0, 1, 1)	$C_{3}-W_{2}-W_{3}+\gamma W_{2}$	$C_{2}+W_{3}-\gamma W_{2}+\theta W_{2}$	$E_{1}-C_{1}-E_{2}-W_{1}$
(1, 1, 1)	$C_{1}-E_{1}+E_{2}+W_{1}$	$C_{3}-W_{1}-W_{2}-W_{3}-\alpha W_{1}+\gamma W_{2}$	$C_{2}+W_{3}-\alpha W_{1}+\beta W_{1}-\gamma W_{2}+\theta W_{2}$

## 5 Educational setting analysis

### 5.1 Setting design

This study simulates various innovation competition scenarios under different educational contexts to further validate the strategy evolution process in the game model. The context design involves the behavioral performance of participants under different policies, innovation atmospheres, and incentive mechanisms, observing how these factors specifically affect innovative behavior by controlling experimental variables.

The five educational settings simulated in this study represent theoretically meaningful scenarios derived from the combinations of active and passive strategic choices made by the three key actors—students, universities, and competition organizers. These scenarios were informed by exploratory interviews conducted with student participants and faculty mentors involved in university-level innovation competitions across Chinese higher education institutions. The aim was not to model specific institutional cases, but rather to construct representative settings that capture the realistic patterns and variations observed in innovation behavior.

#### 5.1.1 Setting 1: passive-passive-passive

**Figure 5:**
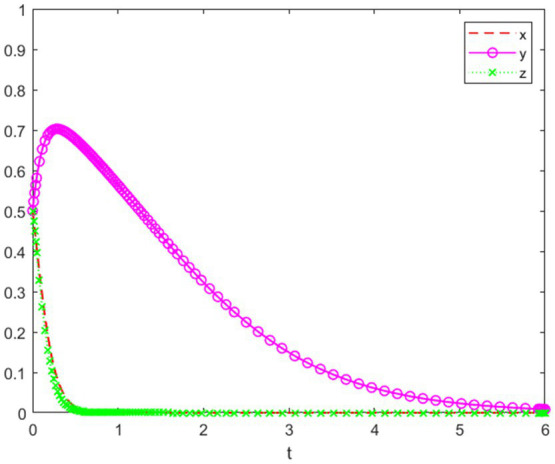
System evolution trajectory under Setting 1.

An innovation competition organized by a company, such as a market research competition, is non-professional in nature. At a key university in China, which is a research-focused institution, the university emphasizes faculty research performance, and students generally have good job market prospects. As a result, most students do not have a strong desire to participate in innovation competitions, and the university does not prioritize supporting such activities. Based on the above analysis, the simulation experiment of the game behaviors of each party is shown in Figure 5.

In Figure 5, the initial willingness of the competition organizers, university, and individual students is set at 0.5, and the system eventually evolves to the equilibrium point (0, 0, 0). The results show that, since the competition organizers and the university did not establish a mandatory mechanism for participation in innovation competitions, the willingness to participate remains low. For students, initially, there is some willingness to engage, but upon realizing that neither the competition organizers nor the university provide corresponding incentives, and that participating in the competition would take up their study time and incur some financial costs, their willingness to participate gradually decreases. Therefore, the strategy choices of the competition organizers and the university have an impact on the strategy choices of individual students.

This setting emphasizes that, in the absence of a comprehensive incentive mechanism, the lack of coordination among all parties negatively influences the psychological behavior of students when it comes to participating in innovation competitions.

#### 5.1.2 Setting 2: passive-active-passive

**Figure 6:**
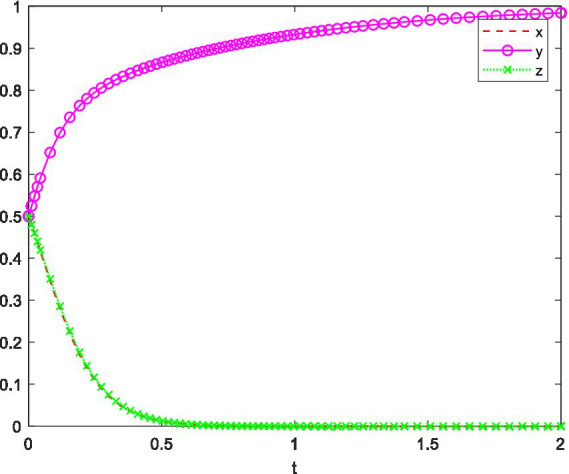
System evolution trajectory under Setting 2.

A company organizes a professional innovation competition for university students, such as the BIM competition. At a key university in China, the Civil Engineering Department, which is a research-oriented institution that prioritizes faculty research performance and does not emphasize student participation in innovation competitions, is used as the setting. However, the BIM competition enhances students’ practical skills, the virtual simulation process attracts students’ interest, and the award certificates from the competition have a significant positive impact on the employability of civil engineering graduates. As a result, students have a strong willingness to participate. Based on the above analysis, the simulation of the strategic behavior of the involved parties is shown in Figure 6.

In Figure 6, the initial willingness of the competition organizers, the university, and the students is set to 0.5, and the system eventually evolves to the equilibrium point (0, 1, 0). The results show that because participating in the competition brings substantial benefits to the students, even though the competition organizers and the university do not have a perfect incentive system, students still have a strong willingness to participate.

This setting emphasizes that, despite the lack of a comprehensive incentive mechanism, the collaboration among the parties has a positive impact on students’ psychological behavior toward participating in innovation competitions, particularly when such participation is highly beneficial to their personal development.

#### 5.1.3 Setting 3: active-active-passive

**Figure 7:**
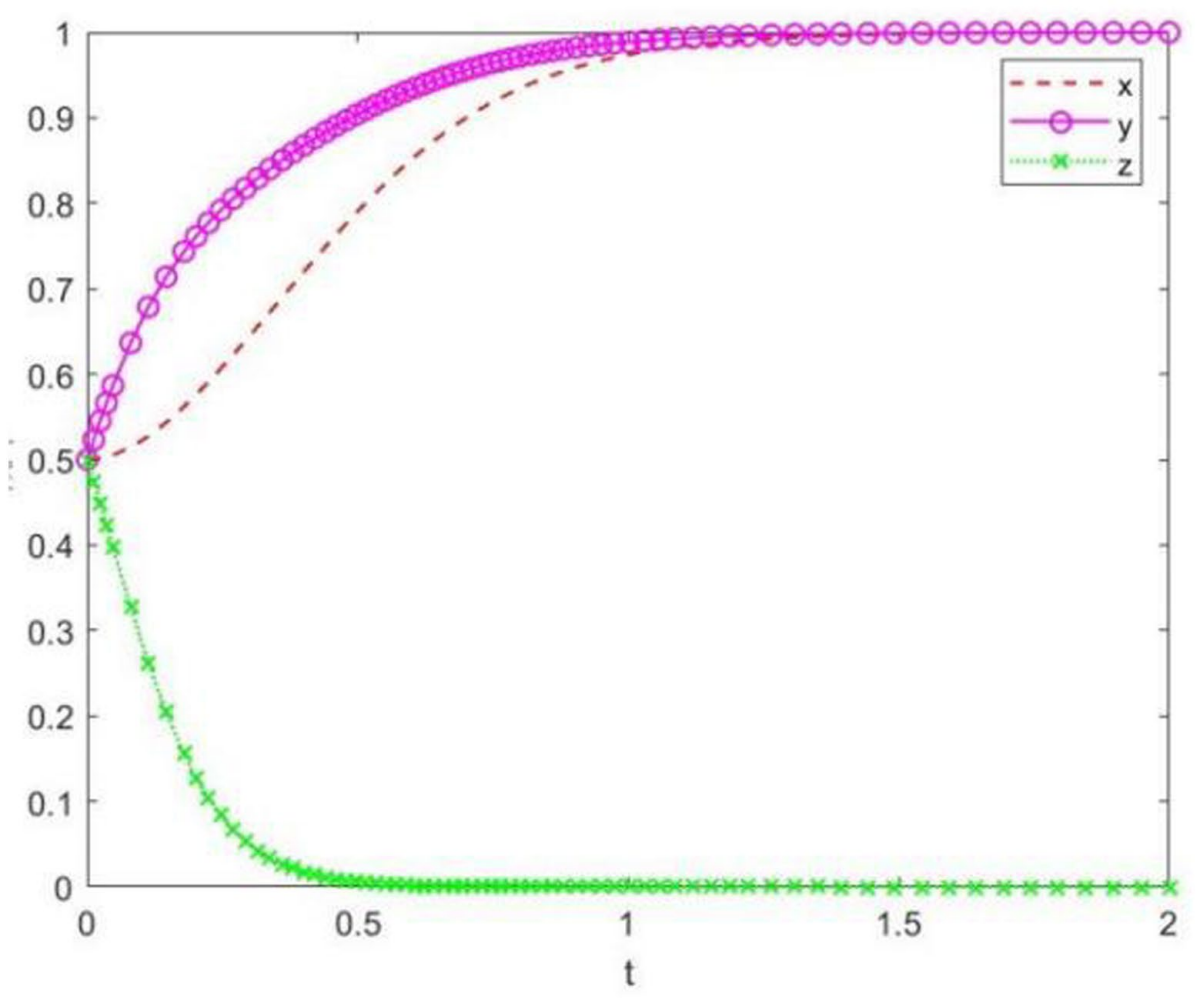
System evolution trajectory under Setting 3.

A government-organized professional innovation competition, such as the “Challenge Cup” competition, is held according to the annual plan. In a certain non-key university in China, the funding is limited. This type of competition is part of the planned expenditure by the relevant government departments, and its implementation results are used as one of the annual assessment indicators for these departments. As a result, the government has established a comprehensive incentive mechanism. For students, the competition covers a wide range of professional fields and is beneficial for employment, which leads to a strong willingness to participate. However, for the university, since many students participate in such competitions and the funding required is substantial, even if the university does not create specific incentive measures, students will still actively participate. Therefore, the university typically does not have a strong willingness to participate. Based on the above analysis, the simulation of the game behaviors among the parties is shown in Figure 7.

In Figure 7, the initial willingness of the competition organizer, the university, and the students are all set to 0.5, and eventually evolve to the equilibrium point (1, 1, 0). The results show that both organizing and participating in the competition bring substantial benefits to the competition organizer and the students. Therefore, even in the absence of specific incentive measures from the university, the organizer will actively host the competition, and the students will still have a strong willingness to participate.

This setting emphasizes that when the organizer is proactive and the university is passive, but the competition is beneficial for the students’ personal development, the collaboration of multiple parties positively influences the psychological behavior of students in participating in innovation competitions.

#### 5.1.4 Setting 4: passive-active-active

**Figure 8:**
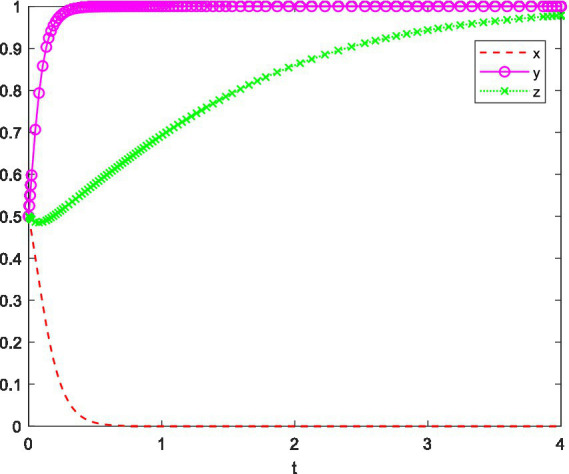
System evolution trajectory under Setting 4.

A professional innovation competition hosted by a company, such as a green building innovation competition. In a certain non-key university in China, the employment situation for students is relatively tough. In terms of scientific research, the university is less prioritized compared to key universities, and in terms of practical skills, it is weaker than vocational and technical colleges. Therefore, the university supports this type of competition, which enhances students’ skills and involves a limited number of participants, providing incentives in terms of funding, credits, and time. As for the students, the competition is beneficial for their future employment, so they have a strong willingness to participate. Based on the above analysis, the simulation of the game behavior of the three parties is shown in Figure 8.

In Figure 8, the initial willingness of the competition organizer, the university, and the individual students is set to 0.5, and the system ultimately evolves to the equilibrium point (0, 1, 1). The results show that the university has developed a relatively comprehensive incentive mechanism based on the development of the university and the employment prospects of the students. The individual students, aiming to improve their practical skills and receiving support from the university in terms of credits, funding, and time, exhibit a strong willingness to participate. As for the company, even without setting up a generous reward system, the university and students’ active participation is enough to encourage the competition, so the company does not adopt superior measures.

This setting emphasizes that when the organizer is passive, the university is active, but the situation is beneficial to the students’ personal development, the collaboration among the parties positively influences the students’ psychological behavior toward participating in the innovation competition.

#### 5.1.5 Setting 5: active-active-active

**Figure 9:**
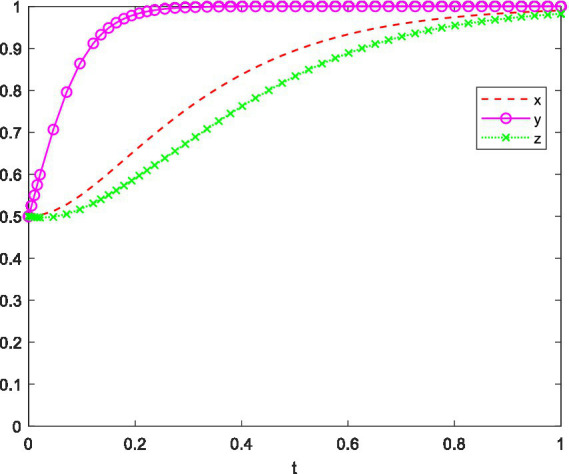
System evolution trajectory under Setting 5.

A specialized innovation competition organized by the government according to the annual plan, such as the “Structural Design” competition. In a non-key university in China, the university considers students’ innovation competition achievements as one of its highlights and features, with a strong atmosphere of student innovation on campus. The relevant government departments (the competition organizers) support the competition for annual performance goals; the university supports the competition based on its educational positioning; and the individual students are highly interested in participating due to their personal development. Based on the above analysis, the simulation of the participants’ game behavior is shown in Figure 9.

In Figure 9, the willingness of all three parties to cooperate gradually increases, forming a positive collaborative pattern, ultimately reaching the equilibrium point (1, 1, 1). The government gains more social benefits by incentivizing innovative behavior, the university benefits and receives rewards by supporting innovation, and individual students gain more overall benefits by engaging in innovation. The system evolution path in this scenario shows a win-win pattern for all parties.

This setting emphasizes that when both the organizers and the university are active and the situation benefits individual student development, the collaboration between all parties positively influences students’ psychological behavior regarding participation in innovation competitions.

### 5.2 Sensitivity analysis

#### 5.2.1 The impact of initial values on evolutionary outcomes

By analyzing the impact of changes in initial values on the evolutionary results, this helps gain a deeper understanding of the relative importance of different variables and parameters in the model. In this study, we use two extreme Settings—Setting 1 and Setting 5—to conduct the analysis.

**Figure 10:**
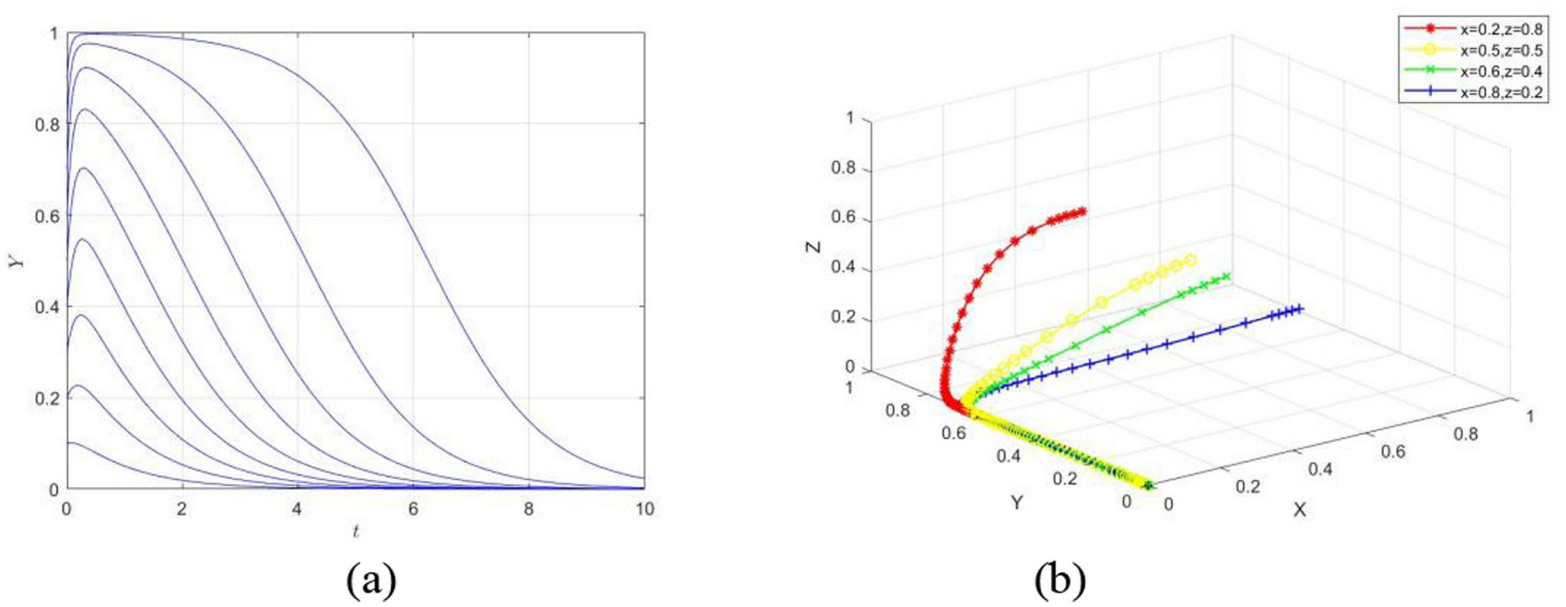
The impact of initial value changes of game participants on students’ psychological behavior in participating in innovation competitions under setting 1. **(a)** Change in initial value of y. **(b)** Change in initial values of x and z.

Firstly, for Setting 1, we analyze the impact of changes in the initial values of the three game participants on the final decision of the individual student, as shown in Figure 10. It can be observed that the evolutionary trajectory of the student’s strategy shows an increasing trend followed by a decrease, ultimately converging to 0. This is because, in the initial stage, the student needs to balance the cost of implementing innovation with personal development. Furthermore, Figure 10b shows that the final decision of the individual student is not influenced by the competition organizer and the university. In Setting 1, the student ultimately chooses the strategy of not actively participating in the innovation competition.

**Figure 11:**
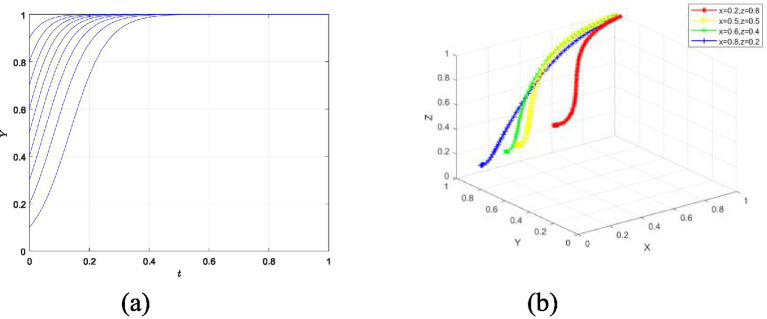
The impact of initial value changes of game participants on students’ psychological behavior in participating in innovation competitions under setting 5. **(a)** Change in initial value of y. **(b)** Change in initial values of x and z.

Secondly, for Setting 5, the impact of initial value changes of the three game participants on the student’s final decision was analyzed. As shown in Figure 11, the final strategy of the student tends to 1. It is particularly noteworthy that the stronger the initial willingness to participate, the faster the student approaches the value of 1. Additionally, Figure 11b shows that the final decision of the student is unaffected by the initial values of the competition organizer and the school, and it ultimately chooses to actively participate in the innovation competition under Setting 5.

#### 5.2.2 The impact of parameter changes on evolutionary outcomes

From the replicator dynamics equation, it can be seen that, in addition to the initial willingness of the game participants, the final evolutionary equilibrium state is also influenced by various parameters in the model. Since Setting 5 represents the stable state pursued by all parties, this case is used to analyze the effect of parameter changes on the outcome.

**Figure 12:**
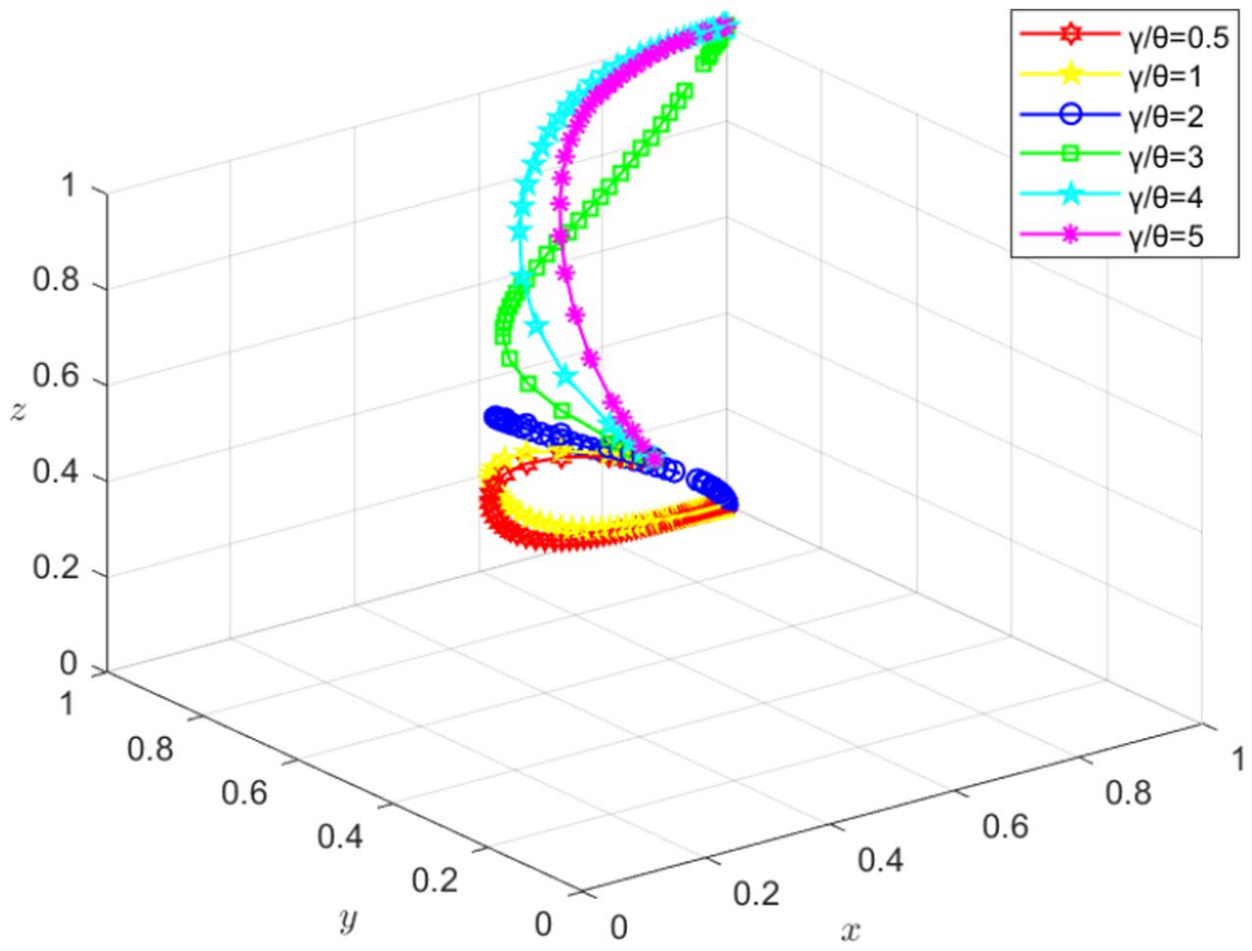
The influence of *γ/θ* on the simulation results of system evolution.

Figure 12 illustrates the impact of the *γ*/*θ* ratio on the system’s evolutionary path. When the γ/θ value changes within the range of 0.5 to 5, the evolutionary trajectories of the three parties can be observed to change and eventually converge to the stable (1,1,1) state. When the γ/θ values are 0.5, 1, and 2, the university ultimately chooses the strategy of not supporting. This suggests that there is a threshold for γ/θ, which directly affects the university’s decision-making. When γ/θ falls below this threshold, the initial willingness of the competition organizer is influenced by the university, leading to a decline in support in the short term. However, with the sustained development of the student’s participation in the competition, the organizer’s overall benefit increases continuously, and the organizer eventually tends to implement an incentive strategy. When γ/θ exceeds this threshold, the strategies of the three parties ultimately converge toward a cooperative stance. Moreover, the higher the γ/θ value, the smoother the system’s evolutionary trajectory, and the faster it converges to the (1, 1, 1) stable state. These results indicate that the differences in the benefits obtained from positive or negative strategies are an important factor influencing whether the university supports students in participating in innovation competitions.

Through the analysis of Setting 5, it is found that parameter changes have a significant impact on the system’s evolutionary outcomes. The support decisions of the competition organizer and the university are influenced by performance benefits and incentive willingness, which, in turn, affect the final cooperative attitude. These factors ultimately influence the psychological changes in students’ participation in competitions. Therefore, when formulating policies and making decisions, it is essential to fully consider the impact of parameter changes on evolutionary outcomes in order to promote cooperation among all parties and foster sustainable development.

## 6 Discussion

### 6.1 Theoretical integration and interpretation

The findings of this study align with key constructs in educational and social psychology. For example, the influence of competition incentives and university policies on student behavior supports the notion of extrinsic motivation as defined in Self-Determination Theory (Ryan and Deci, 2000). The observed sensitivity of students to policy and institutional support also reflects aspects of expectancy-value theory, where students weigh anticipated rewards against perceived costs. Additionally, the role of institutional culture as a moderating factor echoes Bandura’s concept of self-efficacy and social modeling, suggesting that supportive environments can enhance students’ perceived capability to innovate.

### 6.2 Strengths and limitations of the model

A key strength of the tripartite evolutionary game model is its ability to capture dynamic strategic adjustments across stakeholders under varying educational conditions. It provides a flexible framework to simulate behavioral trajectories based on policy and cultural inputs. However, the model also has limitations. First, it assumes bounded rationality and simplified payoff structures, which may not fully reflect the complexity of human decision-making. Factors such as cognitive bias, emotional influence, and peer dynamics are not explicitly modeled. Second, while the five simulated scenarios are grounded in observed trends, they cannot capture the full heterogeneity of real-world institutional contexts. These limitations highlight the need for future empirical validation and possibly the integration of qualitative components such as interviews or survey data.

### 6.3 Implications for theory and educational practice

This study contributes to the integration of educational psychology and behavioral modeling by demonstrating how student innovation behavior is shaped not only by internal cognition but also by dynamic interactions with institutional actors. The proposed model bridges micro-level motivational theory with meso-level structural analysis. For educators and policy designers, the findings emphasize the importance of coordinated support mechanisms across institutions and competition platforms. In particular, sustainable innovation engagement requires not only initial incentives but also reinforcing cultural and policy environments.

In addition, while human resource management (HRM) factors such as faculty development, performance appraisal, and workload distribution play important roles in supporting innovation, the current study focuses specifically on the strategic decision-making mechanisms among students, universities, and competition organizers. Integrating HRM perspectives would add additional layers of complexity, and we acknowledge this as a limitation. Future studies could extend the model by incorporating HRM systems as mediating or moderating variables to better capture institutional dynamics in innovation promotion.

### 6.4 Comparison with existing literature

The empirical results of this study are largely consistent with the conclusions found in existing literature. Cai (2024) pointed out that educational policy support is a key factor in promoting student innovation behavior. Ma and Corter (2019) emphasized the significant impact of campus cultural atmosphere on the cultivation of students’ innovation abilities, while Qu et al. (2024) stressed the role of competition incentive mechanisms in fostering innovation behavior. This study not only validates these traditional viewpoints but also introduces a new perspective by employing a three-party evolutionary game model, which helps better understand the interactions between various factors in the educational environment.

## 7 Conclusions and policy recommendations

### 7.1 Conclusion

This study, based on educational psychology and social psychology, provides an in-depth analysis of the educational environment under the context of innovation competitions for Chinese university students. It explores how educational settings influence university students’ decision-making mechanisms in implementing innovative behaviors. Using a tri-agent evolutionary game model, we reveal the interactive relationships among students, universities, and competition organizers in the decision-making process of innovation behaviors, and validate the profound impact these factors have on students’ psychological willingness to participate in innovation behaviors. The main conclusions of this study are as follows:

First, university policy support, campus culture, and the incentive mechanisms committed by universities play key roles in influencing students’ innovation behavior decisions. Specifically, universities’ investments in the innovation field, such as funding support and innovation projects, significantly enhance students’ willingness and ability to engage in innovation. At the same time, a campus culture with a positive innovation atmosphere can effectively stimulate students’ innovative drive, allowing them to explore new ideas and methods, ultimately improving creativity and practical skills in innovation competition projects.

Second, the incentive mechanisms provided by competition organizers significantly impact students’ psychological willingness to participate in innovation competitions. When competition organizers implement strong incentive mechanisms, students’ motivation to participate increases significantly. This effect is particularly noticeable when the incentives are closely aligned with students’ personal interests, academic goals, and career aspirations. Therefore, well-established incentive mechanisms, such as monetary rewards and employment opportunities, not only increase student participation but also help them translate theoretical knowledge into practical outcomes, thereby enhancing the social and market impact of innovation projects.

Finally, the practical benefits for students’ personal development are crucial considerations in their decision to participate in innovation competitions. Students’ decisions are not only influenced by the external environment but are more driven by the psychological motivation to maximize their personal benefits. When competitions offer tangible career development opportunities and growth prospects, students’ willingness to participate significantly increases, further promoting the participation and effectiveness of innovation competitions. Especially in highly specialized innovation competitions, students can combine theoretical knowledge with practical problems, enhancing their comprehensive abilities and improving employability.

Additionally, it is important to recognize that factors such as the level of a university’s educational positioning, the practical applicability of the competition itself, and students’ overall abilities are key influences on the implementation of innovation competitions. Universities, competition organizers, and individual students interact through the game process, jointly driving students’ innovation behaviors.

Compared to existing literature, this study enriches the theoretical framework of the relationship between the educational environment and students’ innovation behaviors from the perspectives of educational psychology and social psychology, especially within the specific cultural and educational context of China.

### 7.2 Theoretical contribution and practical implications

The study contributes to the intersection of educational psychology and behavioral modeling by integrating motivational constructs (e.g., intrinsic and extrinsic motivation, expectancy-value) into a strategic decision-making framework. It is among the first to use a tripartite evolutionary game model to capture dynamic stakeholder interactions in an educational innovation context. This approach advances current literature by demonstrating how behavioral patterns emerge from system-level feedback loops rather than static variables.

Practically, the findings suggest that universities and competition organizers should design aligned, reinforcing strategies to foster sustainable innovation behavior among students. One-time incentives are insufficient; long-term engagement requires a coherent policy environment and a psychologically supportive culture. For policymakers, this model offers a tool to anticipate behavioral responses to institutional interventions and design more effective innovation ecosystems within higher education.

### 7.3 Policy recommendations

Based on the main findings of this study, we propose the following policy recommendations aimed at optimizing the educational environment and promoting the psychological aspects of university students’ innovative behavior:

- University policy support: Universities should make innovation one of their core tasks and establish long-term, systematic innovation policies. These policies should include providing platforms for student innovation practices, increasing the quantity and quality of innovation-related courses, and offering financial support. Universities should further improve the allocation of research resources, encourage interdisciplinary cooperation, and promote student innovation through practical applications. In addition, universities can set up innovation scholarships or other forms of financial support to motivate students to participate in innovation competitions and projects, thereby enhancing the university’s innovation atmosphere and social impact.
- Campus culture construction: Universities should focus on creating an inclusive, open, and proactive innovation culture. This requires support from the university leadership and active participation from teachers, students, and alumni. Schools can organize regular innovation salons, interdisciplinary lectures, and innovation achievement showcases to provide students with opportunities for communication and display. Additionally, the culture on campus should emphasize a tolerance for failure and an error-friendly environment, encouraging students to boldly experiment with innovations, even if the outcomes are less than ideal. Schools can also further cultivate students’ innovative thinking and practical abilities by guiding them to participate in social practices and innovation-oriented clubs.
- Designing incentive mechanisms by competition organizers: For organizers of various innovation competitions, especially government departments and enterprises, they should design more diversified incentive mechanisms based on students’ needs. These incentives should include not only traditional material rewards (such as prizes and scholarships) but can also extend to entrepreneurial opportunities, industry internships, and career guidance. Moreover, competition organizers should focus on the diversity and fairness of awards to ensure that a wide range of students can benefit from them. To enhance the practical significance and influence of the competition, the organizers should encourage students to translate innovative ideas into tangible results and promote these outcomes to society and the market through relevant platforms, thereby generating greater social value.
- Support from policymakers: Government departments should further improve the policy system for fostering student innovation and provide more resource support for innovation education. In particular, for promising innovative projects, the government can encourage universities and enterprises to invest in innovation education by offering financial subsidies, tax incentives, and other forms of support. Additionally, the government should strengthen guidance and supervision of university innovation activities to ensure the effective implementation of policies, thereby promoting the sustainable development of innovation education.

### 7.4 Future research directions

Although this study provides in-depth theoretical and empirical analysis of the psychological impact of educational contexts on university students’ innovative behavior, there are still several aspects that require further exploration. Future research could expand in the following directions:

- Comparative studies across different cultural and educational contexts: This study focuses on the educational environment and cultural background in China. Future research could extend to other countries and regions to compare the impact of educational environments on university students’ innovative behavior across different cultural contexts. For example, the experience of Western countries in innovation education may differ significantly from that of China, and understanding these differences can help optimize educational policies in a globalized context. Furthermore, research can explore the differences in innovative behavior across different educational systems (such as traditional education versus open education, technical education versus humanities education) to investigate how to enhance students’ innovation abilities within various educational frameworks.
- The relationship between educational environment and innovation outcomes: This study focuses on the impact of the educational environment on students’ innovative behavior. Future research could further explore how the educational environment influences the quality and marketability of students’ innovation outcomes. For example, how improving the organization of innovation competitions can enhance the practical application value of students’ innovative achievements and promote their commercialization and societal integration.
- Longitudinal studies and tracking research: This study primarily constructs a game-theoretic model based on educational psychology and social psychology to analyze the interactions among students, universities, and competition organizers. Future research could conduct long-term tracking studies to observe changes in students’ innovative behavior across different educational stages and career developments. Longitudinal data analysis can provide deeper insights into the sustainability, stability, and long-term impact of the educational environment on students’ innovation behavior.
- The application of educational technology in innovation behavior: With the rapid development of information technology, the application of educational technologies such as online education, virtual innovation competitions, and digital platforms has become an important means to promote innovation education. Future research could explore how educational technologies can provide students with richer innovation resources and collaborative platforms, further enhancing their innovation abilities.

## Data Availability

The original contributions presented in the study are included in the article/supplementary material, further inquiries can be directed to the corresponding author.
